# Results of a 2-Week Inpatient Stay at the Department for Internal and Integrative Medicine: An Observational Study

**DOI:** 10.1155/2012/875874

**Published:** 2012-10-24

**Authors:** Romy Lauche, Holger Cramer, Susanne Moebus, Anna Paul, Andreas Michalsen, Jost Langhorst, Gustav Dobos

**Affiliations:** ^1^Chair of Complementary and Integrative Medicine, University of Duisburg-Essen, 45276 Essen, Germany; ^2^Institute for Medical Informatics, Biometry and Epidemiology, University Hospital of Essen, 45138 Essen, Germany; ^3^Department for Internal and Integrative Medicine, Kliniken Essen-Mitte, 45276 Essen, Germany; ^4^Department of Internal and Complementary Medicine, Immanuel Hospital Berlin, 14109 Berlin, Germany

## Abstract

*Introduction*. The Department for Internal and Integrative Medicine in Essen utilizes mind/body medical elements in order to empower patients with chronic diseases to better cope with their symptoms and to adopt a healthy lifestyle. This study explored the influence and predictors of a 2-week integrative treatment program on patients' quality of life. *Methods*. This observational study was conducted with inpatients as part of the quality assurance program. Patients' quality of life, psychological symptoms, and health locus of control were measured on admission and discharge and again 3, 6, and 12 months after discharge. Regression analyses were conducted to determine the factors predicting improved quality of life. *Results*. Data from 2486 inpatients treated in 2001–2004 were included (80% female, mean age 53.9 ± 14.3 years). Response rates decreased to 50% at 12 months. Small-to-moderate effects were found on patients' quality of life, anxiety, and depression. Patients' internal locus of control significantly increased. Improved quality of life was mainly predicted by lower baseline scores. *Conclusion*. Results of this study suggest that a 2-week inpatient treatment might sustainably reduce patients' symptoms and increase their quality of life; however, conclusions are only preliminary. More research is needed to enable the effectiveness to be judged conclusively.

## 1. Introduction

Integrative treatment approaches are becoming increasingly important. This is especially the case in treating chronic diseases; long-term conditions that develop slowly over time often progress in severity and rarely can be cured [1]. Treating such diseases, including musculoskeletal, cardiovascular, digestive, and metabolic disorders, uses up nearly 80% of nations' health budgets and imposes immense individual burdens [2–4]. Chronic diseases impair physical and mental health and reduce one's ability to carry out activities of daily living [5]. For example, studies have shown that physical illness is an important risk factor for anxiety and depression [6, 7] and that depression is a risk factor for physical illness [8]. 

Although much progress has been made to date in drug development and medical technology, patients and their psychosocial needs are less often involved in the therapeutic process. Despite the effect that psychological health and lifestyle factors are known to have on health and disease in patients with chronic health conditions [9], few change their lifestyles [10]. Empowering patients to adopt healthy lifestyles may enhance the effects of any treatments received and reduce future health risks. 

The Department for Internal and Integrative Medicine at Kliniken Essen-Mitte, the University of Duisburg-Essen's academic teaching hospital, was established in 1999. The Clinic combines conventional medicine, complementary medicine, and Mind/Body therapies to treat patients with chronic diseases [11, 12]. The Mind/Body therapies were added specifically to promote patients' active participation in their care. Mind/Body therapies are defined as “practices that focus on the interactions between the brain, mind, body, and behaviour, with the intention of using the mind to affect physical functioning and promote health” [13]. Such therapies include lifestyle education: seeking to enhance patients' capacity for self-care through such elements as exercise, good nutrition, relaxation, and self-help [12, 14, 15]. Although Mind/Body programs are not overtly psychotherapeutic, aspects of cognitive behavioural therapy are used to enhance patients' ability to cope with their condition, and its impact on daily life, and to live a healthy lifestyle. Research shows such programs' effectiveness for conditions including coronary heart disease [16–18], inflammatory bowel disease [19, 20], and cancer [21]. Patients' control beliefs are also considered key in ensuring long-term treatment effectiveness [22]. Patients who have an internal locus of control (believe that they are able improve their own health) should use active coping strategies more often than other patients. 

This observational study was conducted to explore the influence of a 2-week integrative treatment program on patients' quality of life, psychological symptoms, and locus of control and to determine the factors predicting improved quality of life.

## 2. Methods

### 2.1. Design and Patients

This observational study was conducted at the Department for Internal and Integrative Medicine in Essen [11], Germany, as part of its ongoing quality assurance program. The Clinic was established as a model clinic in 1999 to treat patients with chronic diseases of rheumatological, gastrointestinal, pulmonological, and cardiovascular origin, including those with chronic pain syndromes. Referrals come from specialist and general practitioners, with treatment costs being met by statutory health insurance and many private health insurance companies. The quality assurance program evaluates the Clinic's therapeutic results and cost-effectiveness on behalf of the North Rhine-Westphalia federal state government. Previous partial publication of these results [16, 22] omitted the first year of evaluation [22] and specific patient subgroups [23]. Data on the Clinic's cost-effectiveness are not reported here.

All patients admitted to the Clinic between January 2001 and January 2004 received detailed study information and were invited to participate in this study. Patients who were willing to participate signed informed consent forms. Participants received questionnaires on their admission to (ADM) and discharge from (DIS) a 2-week inpatient hospital stay, with further questionnaires at 3-(FU3), 6-(FU6), and 12 month (FU12) intervals after discharge.

### 2.2. Intervention

Patients received two weeks of integrative inpatient hospital treatment; following individual treatment plans developed from extensive anamneses by physicians, nurses, and mind/body therapists. Treatments included conventional diagnostic and interventional medical approaches, including physiotherapy, and the use of complementary techniques. The latter included the use of traditional medicine (Traditional Chinese Medicine, acupuncture, cupping, leeching, etc.) and classical naturopathy (hydrotherapy, thermotherapy, manual therapy, massage, phytotherapy, exercise, nutritional therapy, and fasting). Patients also received several mind/body therapy sessions, focusing on exercise, stress-reduction, diet, and self-help, to empower them to adopt healthy lifestyles. These sessions were based on Harvard Medical School's Benson-Henry Institute for Mind/Body Medicine Program [24] and the University of Massachusetts' Mindfulness-Based Stress Reduction Program [25, 26]. Elements of cognitive restructuring were also added in this study [27, 28].

### 2.3. Outcome Measures

The following were used to evaluate patients' postintervention change. 

### 2.4. Primary Outcome Measure

#### 2.4.1. Health Related Quality of Life (SF-36)

Patients' health-related quality of life was assessed using the short form 36 of the health survey questionnaire (SF-36) [29]. This tool measures individuals' quality of life on eight dimensions and two main component scales (physical, mental). It has proven validity and reliability [29]. Each scale ranges from 0 to 100, with higher scores indicating higher quality of life. In this study, only the main component summaries were analysed; comparing the outcome with age- and gender-matched values from the German general population [30, 31]. The differences found are presented as *z*-scores, with 0 representing the mean and 1 the standard deviation of the population. The SF-36 assesses quality of life by means of daily living activities, making its use inappropriate in a hospital setting. The SF-36 was, therefore administered at ADM, FU3, FU6, and FU12, but not at discharge, in this study.

### 2.5. Secondary Outcome Measures

#### 2.5.1. Anxiety and Depression (HADS)

Changes in patients' psychological symptoms were measured using the Hospital Anxiety and Depression Scale (HADS). This tool has 14 items, scored on 4 point Likert scales [32]. Higher scores indicate more severe symptoms. For both dimensions, cut-off scores have been introduced to indicate possible sub-syndromal (≥8) or clinically relevant (≥11) anxiety or depression [33]. Study patients completed the HADS at admission, discharge, and followup.

#### 2.5.2. Health Locus of Control (GKÜ)

Patients' health locus of control was measured using the GKÜ (German abbreviation for Gesundheitsbezogene Kontrollüberzeugungen) [34, 35] a short version in German of the Multidimensional Health Locus of Control Questionnaire (MHLC) [36]. The GKÜ is a 9-item questionnaire which assesses three dimensions of patients' control beliefs: internal, external-social, and external-fatalistic. The more internal control patients perceive, the more they feel able to influence their health. By contrast, the more control that patients attribute to others (external-social), or to luck or destiny (external-fatalistic), the less they feel able to influence their health. The GKÜ was originally devised for use with patients with cancer, but has since been adapted for use with other patients, such as those with back pain [37]. A previous study reported problems with one item on the GKÜ [38]. A factor analysis undertaken with the present study data showed that this item loaded on two factors equally. It was therefore deleted from the analysis [38]. Patients completed the GKÜ at admission, discharge, and followup.

#### 2.5.3. Satisfaction with Health and Life in General (FLZ)

Patients' satisfaction with their health and lives in general was measured using two 5-point Likert items from the Life Satisfaction Questionnaire (Fragebogen zur Lebenszufriedenheit, FLZ) [39] at admission, discharge, and followup. The scale used ranged from 1 = very dissatisfied to 5 = very satisfied, with higher scores indicating patients' greater satisfaction with their health and lives in general.

#### 2.5.4. Demand on Medical Services

The study questionnaire also asked patients to record how many doctors' visits they had made during the previous month. Patients were also asked, at admission and 12-month followup, how many days they had been admitted for hospital inpatient treatment and how many days they had missed work (where appropriate) over the previous year. 

#### 2.5.5. Improvement in Disease-Related Symptoms

At discharge and each followup, patients compared their main disease-related symptoms with before they entered treatment, using a 5-point Likert scale that ranged from “much worse” to “much improved.” 

#### 2.5.6. Statistical Analysis

All analyses were based on full data sets with no missing data. Baseline comparisons and comparisons between responders and non-responders were conducted using *t*-tests for independent groups, for parametric data, and *x* ^2^-tests for nonparametric data. Responders were defined as patients who returned their questionnaires at given time points, with nonresponders being those who failed to do so.

Repeated analyses of variance (ANOVA) were used to analyse the study's primary and secondary outcome measures as appropriate. Residuals were checked visually for normal distribution. The homogeneity of variance was tested using a Mauchly test for sphericity. Cases of nonsphericity were corrected using a Greenhouse-Geisser correction. The estimated differences between the time points, from the ANOVA analyses, and their 95% Confidence Intervals, were reported for each outcome. Effect size Cohen's d (the estimated differences from the ANOVA analyses divided by the standard deviation of patients' admission scores) was also given. Changes in patients' disease-related symptoms were explored solely descriptively.

Analyses were conducted in the following order to determine the factors influencing the study's primary outcome measure: (1) bivariate correlations were used to explore possible links between patients' sociodemographic characteristics, their scores at admission and their outcome at discharge and followup. (2) Factors with significant correlations (*r* ≥ 0.1) were entered as possible predictors into the regression analysis. Patients' age and gender were automatically included in each regression. (3) Linear forward stepwise regression analysis with linear outcome and linear or dichotomous regressors was then conducted. 

All analyses were conducted using the Statistical Package for the Social Sciences (SPSS) (Version 20.0, IBM, USA). The significance level was set at *α* = 5%. An automatic Bonferroni correction was applied for the posthoc comparison of the main ANOVA effects.

## 3. Results

### 3.1. Patients' Characteristics

**Figure 1:**
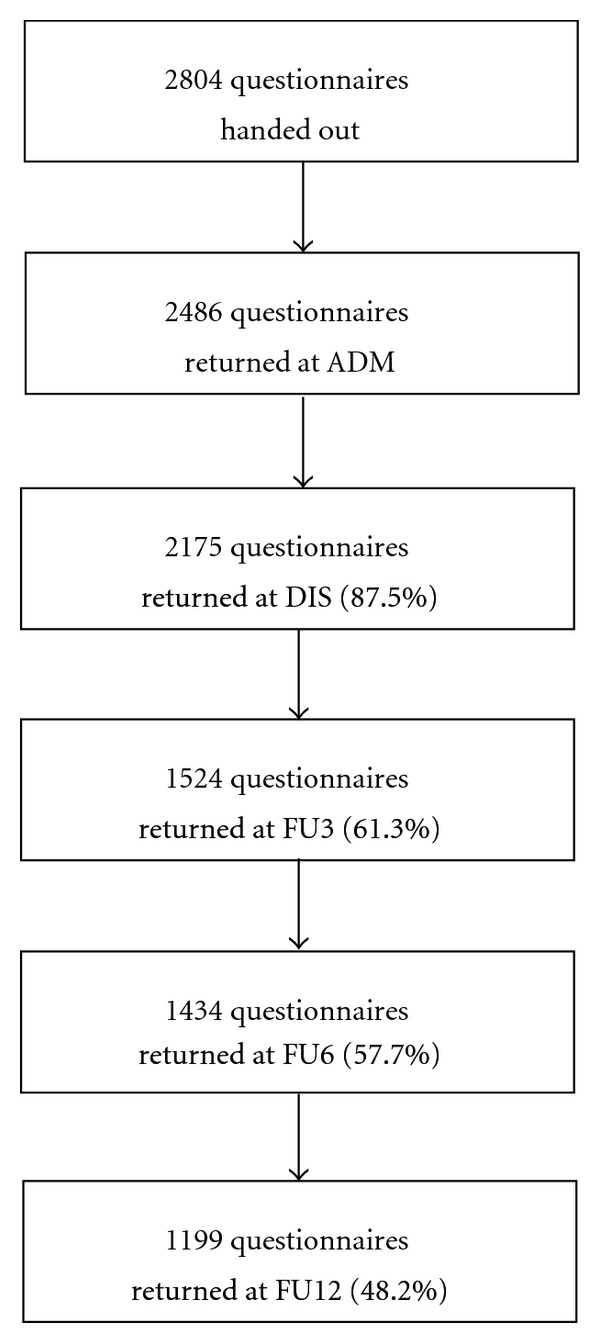
Flowchart of patient response rates.

The study's response rates are shown in Figure 1. Of the 2804 patients treated between January 2001 and January 2004, 2486 agreed to participate in the study on admission. The initial response rate of 87.5% on discharge, diminished to 61.3%, 57.7%, and 48.2% at 3, 6 and 12 months' followup. The numbers of patients included in the analyses are shown in each of the following tables. Since only complete patients' data sets were included in the analyses, a missing score at any time point led to exclusion of this patient's data.

Table 1 shows patients' sociodemographic and clinical characteristics on admission. The study sample consisted mainly of women in their mid-50s; most of whom had not been educated to A-level standard. Men more often reported being in a relationship, with more education, more frequent absenteeism from work over the past year and lower expectations of improvement from their inpatient stay than women. 

More than two thirds of the patients experienced a chronic pain condition, with back pain, headache, fibromyalgia and arthritis being the most frequently cited causes for admission. More than half of the study patients were diagnosed as severely affected by their disease conditions, with few being seen as only slightly affected.

Most patients had high expectations of complementary medicine's effectiveness, with more than 80% expecting some improvement and few expecting none. 

The following results compare responders' and nonresponders' sociodemographic and clinical characteristics to indicate potential response bias, illustrate the primary and secondary outcomes for patients with complete data sets, and present the regression analysis, for patients with complete data sets, to determine the factors associated with improvements in their quality of life after inpatient treatment. 

### 3.2. Comparison of Responders and Nonresponders

An eighth of patients (12.5%) were lost to followup between admission and discharge. More than a third (38.7%) had withdrawn within three months of discharge (FU3), the time of the biggest withdrawal.  Table 2 shows the significant differences found between responders and nonresponders.

Table 2 shows that responders stayed longer in hospital, experienced less psychological symptoms, and scored more highly on the mental health component summary of the SF-36 than non-responders. Responders' levels of internal locus of control were also higher at baseline, and their external-fatalistic control beliefs lower, than nonresponders'. Three months posttreatment, responders' results also outstripped non-responders' with regard to their satisfaction with their life in general and perceived improvements in their symptoms at discharge.

### 3.3. Primary Outcome Measure

#### 3.3.1. Health-related Quality of Life (SF-36)

The physical (PCS) and mental component (MCS) summaries of patients' SF-36 scores increased from admission to FU3; remaining relatively stable thereafter, see Table 3. Repeated ANOVA analyses showed a significant time effect on both the PCS (*P* < 0.0001) and the MCS (*P* < 0.0001). Patients' physical (PCS) and mental component summaries (MCS) were found to be significantly higher at FU3, FU6 and FU12, when compared to admission. No statistical differences were found between patients' follow-up measurements. The effect sizes for patients' PCS and MCS summaries were small to moderate. Altogether 41.3% and 44.3% had increased their PCS and MCS summaries respectively by more than five points from discharge onwards; a clinically important improvement in their quality of life [29].

Compared to age- and gender-matched data from a normative German population, patients' PCS was *z* = −1.49 and their MCS *z* = −0.97 at admission; below the average of the corresponding norms. At FU3, study patients' PCS was *z* = −1.01 and their MCS = −0.47; an improvement of 0.5z.

### 3.4. Secondary Outcome Measures

#### 3.4.1. Anxiety and Depression (HADS)

ANOVA analyses revealed significant time effects on patients' anxiety (*P* < 0.0001) and depression (*P* < 0.0001) scores. Post-hoc analysis found patients' scores to be significantly lower on discharge and at followup than on admission. Although HADS-A and HADS-D increased on followup, they were still significantly lower than on admission. The effect sizes for anxiety and depression were moderate on discharge and small at followup, see Table 3.

The proportion of patients with cut-off scores >8 (indicating subsyndromal anxiety or depression) and >11 (indicating a clinically relevant disorder) were relatively high on admission (HADS_A ≥ 8: 61.1%; HADS_A ≥ 11: 35.5%; HADS_D ≥ 8: 46.5%; HADS-D ≥ 11: 22.4%). These scores were lower on both discharge (HADS_A ≥ 8: 34.5%; HADS_A ≥ 11: 14.3%; HADS_D ≥ 8: 22.0%; HADS_D ≥ 11: 9.4%) and followup. At FU12, 33% less patients showed signs of subsyndromal or clinically relevant anxiety or depression, compared to admission (HADS_A ≥ 8: 42.3%; HADS_A ≥ 11: 22.1%; HADS_D ≥ 8: 31.9%; HADS_D ≥ 11: 16.8%).

#### 3.4.2. Health Locus of Control (GKÜ)

Significant changes were found for all three scales of the GKÜ over time (all *P* < 0.001). Discharge and follow-up scores were significantly higher for internal and significantly lower external-social control beliefs, compared to admission (see Table 3). Follow-up scores were also higher for external-fatalistic beliefs.

The ratio of patients' internal to external control beliefs, suggesting a perceived shift in control beliefs, also increased significantly over time (*P* < 0.001), with all follow-up scores being significantly higher than on admission.

#### 3.4.3. Satisfaction with Health and Life in General (FLZ)

Patients' satisfaction scores are shown in Table 3. ANOVA analysis showed significant time effects (*P* < 0.001), with increases in satisfaction with health found throughout the follow-up period, compared to baseline. Patients' satisfaction with their lives in general was higher than their satisfaction with their health, on average. The ANOVA results also showed significant time effects for patients' satisfaction with their lives in general (*P* < 0.001), with significant differences found between baseline and all follow-up measurements. The increase in the latter, however, was only marginally.

#### 3.4.4. Demand on Medical Services

In the posttreatment year (FU12), employed patients' absenteeism from work fell by some 10 days (*P* < 0.0001). Patients' visits to doctors in the previous month were also significantly reduced (*P* < 0.0001). No significant change occurred in the number of nights that patients spent in hospital during this year.

#### 3.4.5. Improvement of Disease-Related Symptoms

On discharge, almost 80% of the study patients rated their health as at least somewhat improved, with some 60% continuing to do so 12 months later. In contrast, at FU12, a fifth of patients (20%) saw their health as worse than before admission.

#### 3.4.6. Predictors of Improvement in the Primary Outcome Measure

All of patients' socio-demographic and clinical admission data with significant correlations of *r* ≥ 0.1 were entered into the predictor analysis, to determine the factors influencing the changes found in their SF36 physical (PCS) and mental (MCS) component summaries at 3-month followup. The resulting analysis showed that change in patients' PCS score at FU3 was predicted by their PCS score on admission (*β* = −0.33), their internal control beliefs on admission (*β* = 0.13), age (*β* = −0.11), their MCS score on admission (*β* = 0.13), a main diagnosis of a pain versus a nonpain condition (*β* = −0.09) and being employed (*β* = 0.09) (all *P* < 0.05). Patients' MCS at FU3 was predicted by their MCS score on admission (*β* = −0.55), a higher educational level (*β* = 0.07), and their satisfaction both with life in general (*β* = 0.15), and with health (*β* = 0.06) on admission (all *P* < 0.05). 

## 4. Discussion

### 4.1. Summary of the Results

This observational study investigated changes occurring in patients with chronic conditions following a 2-week inpatient integrative program. The latter combined conventional medicine, complementary medicine and Mind/Body therapies aimed at empowering patients to adopt a healthy lifestyle. The results revealed a small-to-moderate, but sustained improvement in patients' perceived quality of life, with regard to both its physical and mental health aspects. Patients also reported reduced anxiety and depression, as measured on the Hospital Anxiety and Depression Scale (HADS). At the same time, patients' internal locus of control increased and their external-social control beliefs diminished. On the other hand, patients' external-fatalistic control beliefs rose significantly over time. Patients' satisfaction with their health and lives in general increased. Employed patients reported fewer days' absenteeism and all patients made fewer doctors' visits during the posttreatment year. Altogether, patients reported substantial benefit from their two weeks of integrated inpatient treatment.

### 4.2. Comparisons with the Literature

The above results are in line with those of other studies into the effectiveness of inpatient treatments that include naturopathy and Traditional Chinese Medicine [40–48]. Patients in these other observational trials also showed improved symptoms and health, as well as improved quality of life. The effect sizes were also comparable between these studies, for example, Melchart et al. [47] found effects around *d* = 0.5 for changes to patients' PCS and MCS after 6 months, changes only marginally larger than those found in the present study. More than two fifths of the patients in the present study (42%) reported improvements of >5 points in the SF-36, which is considered clinically relevant [49]. The effect sizes for the HADS anxiety and depression scales were moderate on discharge and small on followup, although the frequency with which patients scored above the cut-offs for subsyndromal and clinically relevant anxiety and depression each fell by almost a third (33%).

Patients with chronic conditions often suffer from comorbid psychological disorders. Falls in the levels of anxiety and depression that patients reported in this study, following their inpatient treatment may reflect improvements in their overall health. They may also reflect the impact of the psychosocial approaches used in the Mind/Body therapy element of the treatment received. 

The regression analysis conducted showed that the change in the SF-36 physical component summary was predicted by patients' PCS score on admission. Patients with lower PCS scores on admission benefited most, perhaps because they began with very low scores and thus had more room to improve. The effect might also reflect a regression to the mean.

For the MCS at baseline, the link was reversed. The higher patients' MCS scores were on admission, the more their PCS scores improved at followup. Patients' pain diagnoses, age, and employment status also proved important in this study. Patients who had nonpain diagnoses were younger and were employed were more responsive, perhaps because they had shorter disease histories and were potentially less impaired than those unable to work. Comparable results have been found for patients with low back pain [50, 51]. Together, these variables explained 12.4% of the variance found, suggesting minor predictive power. 

For improvements in patients' MCS scores, lower MCS scores at admission, higher levels of satisfaction with health and life in general and a higher level of education proved significant. Higher degrees of satisfaction might be linked to greater levels of openness and appreciation which, together with higher levels of education, might incline patients towards psychosocial Mind/Body interventions. All of the selected factors, taken together, explained 24.4% of the MCS change variance found. 

An internal locus of control proved the only significant factor predicting change in patients' PCS scores, but did not influence their MCS scores. Improvements in patients' physical health were perhaps linked to an increased probability of adopting a healthy lifestyle in patients with a more internal locus of control. Adequate exercise, nutrition, and relaxation are certainly considered important to overall health and mortality [52–54]. Further research is needed to explore the link between patients' locus of control and their actual behaviours, given that neither external-social nor external-fatalistic control beliefs predicted any study outcomes. Although patients' external-fatalistic control beliefs increased during inpatient treatment, which was contrary to expectations, this change may reflect the questionable discriminative power of the tool used. The Mind/Body program offered at the Department for Internal and Integrative Medicine, based on similar programs used elsewhere, draws on patients' acceptance of their health situations. Accepting patients might be mistakenly seen to have high external-fatalistic control beliefs because the questionnaire used cannot distinguish between these two variables. The study data provide no conclusive evidence about this matter. Further studies are therefore needed to evaluate the relationship between patients' locus of control and their acceptance of their health situations. Data on patients' posttreatment demands on medical services was also inconclusive, although there was some suggestion of falls in absenteeism and the number of doctors' visits made.

### 4.3. Limitations of the Study

This study's findings are weakened by its observational design; the lack of a control group renders the effects indivisible from nonspecific effects. There may also have been some selection bias, as 11.3% of all treated patients chose not to participate in the study. Another problem resulted from the followup response rate the loss of 50% of the patients at 12 months generating much missing data. Whilst other quality assurance programs have experienced similar response rates [41, 42], some have lost only 25% of their participants [47]. The followup losses experienced in the present study may mean that the reported results reflect a bias in patients' response behaviours. To detect such a bias, responders' and nonresponders' socio-demographic and baseline characteristics were compared. Non-responders were found to have shorter clinic stays (potentially reducing delivery of the study discharge questionnaire), more external-fatalistic control beliefs, higher depression scores and less mental health than responders. They also cited less symptomatic improvement on discharge then responders. Taken together, these comparisons suggest that patients with more disadvantageous control beliefs, greater psychological symptoms and less benefit from treatment were more often lost to followup. The reported effects might therefore be an overestimation and, in part, reflect regression to the mean. 

Since neither the patients nor the program has changed for the most part, these data, retrieved about 10 years ago, are still valid and the results generalizable to current conditions. The Clinic still provides internal and external quality assurance program, but with other quality indices.

### 4.4. Future Research

This study's results suggest that patients experience benefit from receiving two weeks of integrative inpatient treatment, but more research is needed to confirm and extend them. Future studies should use more rigorous designs, such as randomised controlled trials, to explore links between patients' sociodemographic, clinical characteristics and selected outcome measures, delving into the nature of and mechanisms behind these links. They should also focus on ways to enhance patient compliance.

## 5. Conclusion

The results of this study suggest that a 2-week inpatient treatment might sustainably reduce patients' somatic and psychological symptoms and increase their quality of life; however, conclusions are limited by the observational study design and a high withdrawal rate. Increases in patients' quality of life were predicted by several variables; the most important being low quality of life at admission. More research is needed for conclusive judgment of integrated inpatient treatment programs' effectiveness and modes of action.

## Figures and Tables

**Table 1:** Sociodemographic and clinical data of patients at admission.

		All patients	Female	Male	*P*
Complete sample size		2486	1994	492	
Age (M ± SD; range)	(*N* = 2486)	53.9 ± 14.3; 16–91	53.87 ± 14.3	54.8 ± 14.5	0.77
Family status (% in relationship/married)	(*N* = 2438)	58.1	54.9	71.2	<0.001
Education (% with A-level and higher)	(*N* = 2414)	26.3	24.0	35.8	<0.001
Number of doctors consultations within the past 4 weeks (M ± SD; range)	(*N* = 2241)	3.9 ± 3.4; 0–25	3.8 ± 3.2	4.2 ± 3.9	0.06
Days of sick leave in the past 12 months (M ± SD; range)	(*N* = 881)	25.9 ± 56.3; 0–365	23.6 ± 49.2	33.3 ± 74.7	0.03
Days admitted to hospitals within the past year (M ± SD; range)	(*N* = 914)	21.4 ± 19.3; 2–70	21.2 ± 20.0	22.2 ± 19.1	0.53
Length of stay at the hospital in days (M ± SD; range)	(*N* = 2486)	14.6 ± 3.6; 4–54	14.7 ± 3.6	14.1 ± 3.4	0.001
Expectancy of improvement (%)	(*N* = 2398)				
(i) Very much		44.8	48.2	31.7	<0.001
(ii) Somewhat		37.4	35.5	45.0	
(iii) Unsure		16.6	15.3	21.7	
(iv) Not so much		1.0	0.7	1.9	
(v) Not at all		0.3	0.4	0.0	
Major admission diagnosis (%)	(*N* = 2486)				
(i) Arthritis		6.7	7.2	4.7	
(ii) Fibromyalgia		10.8	13.0	2.0	
(iii) Headache		11.3	12.1	7.7	
(iv) Hypertension		3.4	2.7	6.3	
(v) IBD (Crohn, Colitis)		4.9	3.9	9.3	
(vi) IBS		3.0	3.3	1.6	
(vii) Ischemic cardiac disease		1.2	0.5	4.1	
(viii) Lung diseases		5.5	5.7	4.5	
(ix) Osteoarthritis		9.9	10.5	7.3	
(x) Spinal Pain		18.8	18.2	21.5	
(xi) Pain, others		10.9	10.5	12.4	
(xii) Others		13.7	12.5	18.5	
Physician rated severity of disease (%)	(*N* = 2486)				
Very severe		6.5	6.8	5.3	
Severe		49.3	52.4	36.7	
Moderate		39.1	37.2	46.8	
Slight		4.2	3.1	8.7	
Minor		0.9	0.5	2.5	

M: mean; SD: standard deviation. For several variables, data were inconclusive. For work absenteeism, only scores from patients with full-time or part-time employment were entered.

**Table 2:** Significant differences at baseline characteristics and health change at discharge between responders and non-responders.

Characteristic	Responders at DIS (M ± SD)	Nonresponders at DIS (M ± SD)	*P*
Length of stay at the hospital (in days)	14.8 ± 3.2	13.3 ± 5.5	<0.001
Internal control beliefs (GKÜ)	3.0 ± 0.9	2.9 ± 0.9	0.034
External-fatalistic control beliefs (GKÜ)	2.2 ± 0.9	2.4 ± 0.9	0.001
Depression (HADS)	7.4 ± 4.2	8.1 ± 4.2	0.01

Characteristic	Responders at FU3 (M ± SD)	Nonresponders at FU3 (M ± SD)	*P*

Length of stay at the hospital (in days)	14.8 ± 3.0	14.3 ± 4.4	<0.001
External-fatalistic control beliefs (GKÜ)	2.2 ± 0.9	2.2 ± 0.9	0.01
External-social control beliefs (GKÜ)	2.8 ± 1.0	2.9 ± 1.1	0.035
Depression (HADS)	7.3 ± 4.1	7.9 ± 4.3	<0.001
Mental component summary (SF-36)	40.2 ± 12.9	38.7 ± 12.6	0.004
Satisfaction with life in general	3.4 ± 0.9	3.3 ± 0.9	0.01
Subjective health change at discharge (in %)			0.001
(i) Much improved	33.2	25.7	
(ii) Somewhat improved	47.4	52.4	
(iii) The same	15.8	16.5	
(iv) Somewhat worse	2.7	3.9	
(v) Much worse	0.9	1.5	

DIS: discharge; FU3: 3-month followup; M: mean; SD: standard deviation.

**Table 3:** Pre- post- and follow-up scores, estimated differences, and effect sizes for study outcomes.

Outcome (Questionnaire)	No. of patients^§^	Outcomes (M ± SD)					Estimated differences* to admission (M; 95% CI)				Estimated effect sizes^#^ (Cohen's d)			
		ADM	DIS	FU3	FU6	FU12	DIS	FU3	FU6	FU12	DIS	FU3	FU6	FU12
Quality of life														
Physical component summary (SF-36)	(*N* = 796)	32.9 ± 10.2		37.4 ± 11.7	37.4 ± 12.1	38.0 ± 12.3		5.0; −4.4 to 5.6	4.2; 3.5 to 4.8	4.6; 3.9 to 5.2		0.49	0.41	0.45
Mental component summary (SF-36)	(*N* = 796)	39.6 ± 12.8		44.7 ± 12.6	44.8 ± 12.4	45.1 ±12.7		4.9; 4.1 to 5.6	4.9; 4.0 to 5.7	4.6; 3.8 to 5.4		0.38	0.38	0.36

Psychological symptoms														
Anxiety (HADS_A)	(*N* = 850)	9.0 ± 4.3	5.7 ± 3.9	6.9 ± 4.4	6.8 ± 4.5	6.8 ± 4.6	−2.9; −3.2 to −2.5	−1.7; −2.0 to −1.3	−1.8; −2.2 to −1.4	−1.8; −2.2 to −1.4	−0.66	−0.39	−0.41	−0.41
Depression (HADS_D )	(*N* = 853)	7.1 ± 4.1	4.4 ± 3.6	5.8 ± 4.3	5.8 ± 4.4	5.9 ± 4.4	−2.7; −3.0 to −2.4	−1.2; −1.6 to −0.9	−1.3; −1.6 to −0.9	−1.2; −1.5 to −0.8	−0.66	−0.30	−0.31	−0.29
Satisfaction with health (FLZ)	(*N* = 865)	2.2 ± 0.9	—	2.7 ± 1.0	2.7 ± 1.0	2.8 ± 1.0	—	0.5; 0.4 to 0.6	0.6; 0.5 to 0.7	0.6; 0.5 to 0.7		0.54	0.59	0.60
Satisfaction with life in general (FLZ)	(*N* = 868)	3.5 ± 0.9	—	3.6 ± 0.9	3.6 ± 0.9	3.6 ± 0.9	—	0.2, 0.1 to 0.2	0.1; 0.1 to 0.2	0.2; 0.1 to 0.2		0.18	0.15	0.17

Control beliefs														
Control belief internal (GKÜ)	(*N* = 893)	3.0 ± 0.9	3.6 ± 0.9	3.3 ± 0.9	3.3 ± 0.9	3.3 ± 1.0	0.6; 0.5–0.6	0.4; 0.3–0.4	0.3; 0.3–0.4	0.3; 0.2–0.4	0.66	0.41	0.36	0.34
Control belief external-social (GKÜ)	(*N* = 881)	2.8 ± 1.0	2.7 ± 1.0	2.6 ± 1.0	2.6 ± 1.0	2.6 ± 1.0	−0.1; −0.2 to −0.1	−0.2; −0.2 to −0.1	−0.2; −0.2 to −0.1	−0.2; −0.2 to −0.1	−0.14	−0.16	−0.16	−0.17
Control belief external-fatalistic (GKÜ)	(*N* = 877)	2.1 ± 0.8	2.1 ± 0.8	2.2 ± 0.9	2.2 ± 0.8	2.3 ± 0.8	0.0; 0.0 to 0.0	0.1; 0.1 to 0.2	0.1; 0.1 to 0.2	0.2; 0.1 to 0.2	0.02	0.16	0.16	0.22
Ratio internal/external (GKÜ)	(*N* = 876)	1.3 ± 0.8	1.6 ± 1.0	1.6 ± 1.0	1.6 ± 1.0	1.6 ± 1.0	0.4; 0.3 to 0.4	0.3; 0.2 to 0.3	0.3; 0.2 to 0.3	0.3; 0.2 to 0.4	0.47	0.37	0.39	0.40

Demand of medical services														
Days of sick leave	(*N* = 264)	18.9 ± 36.0				8.3 ± 13.1				−10.5; −14.9 to −6.1				−0.26
Number of doctors' visits	(*N* = 705)	3.7 ± 3.2		2.3 ± 2.5	2.5 ± 3.0	2.8 ± 3.1		−1.4; −1.7 to −1.2	−1.3; −1.5 to −1.0	−1.0; −1.3 to −0.7		−0.46	−0.40	−0.30
Days admitted to a hospital	(*N* = 202)	23.2 ± 20.3				25.9 ± 21.3				2.7; −0.8 to 6.1				0.13

Subjective health change (%)														
(i) Much improved			30.8	24.8	23.7	27.9								
(ii) Somewhat improved			49.0	38.3	35.4	31.3								
(iii) The same			16.0	23.7	23.4	22.1								
(iv) Somewhat worse			3.1	7.0	8.8	9.8								
(v) Much worse			1.1	6.1	8.8	8.9								

ADM: admission; DIS: discharge; FU3: 3-month follow-up; M: mean; SD: standard deviation; CI: confidence interval; *estimation from repeated measurement ANOVA. ^#^Effect size calculation was based on estimated differences. ^§^The number of patients included in the analyses may be smaller due to missing data at any measurement time-point, leading to their exclusion within the repeated measurement ANOVA.
